# Early onset recall pneumonitis during targeted therapy with sunitinib

**DOI:** 10.1186/1471-2407-13-3

**Published:** 2013-01-02

**Authors:** Takeshi Yuasa, Shinichi Kitsukawa, Gen Sukegawa, Shinya Yamamoto, Keita Kudo, Kazunari Miyazawa, Takuyo Kozuka, Sohei Harada, Junji Yonese

**Affiliations:** 1 Department of Urology, Cancer Institute Hospital, Japanese Foundation for Cancer Research, Ariake, Tokyo, 135-8550, Japan; 2 Department of Thoracic Medical Oncology, Cancer Institute Hospital, Japanese Foundation for Cancer Research, Ariake, Tokyo, 135-8550, Japan; 3 Department of Radiation Oncology, Cancer Institute Hospital, Japanese Foundation for Cancer Research, Ariake, Tokyo, 135-8550, Japan; 4 Department of Infectious Diseases, Cancer Institute Hospital, Japanese Foundation for Cancer Research, Ariake, Tokyo, 135-8550, Japan

**Keywords:** Sunitinib, Renal cell cancer, Recall pneumonitis, Radiation, Radiation pneumonitis

## Abstract

**Background:**

Sunitinib interacts with radiation therapy, leading to synergism of the toxicities of these treatments. Radiation recall pneumonitis is a rare but serious complication of targeted therapy with tyrosine kinase inhibitors.

**Case presentation:**

The case of a patient with metastatic renal cell cancer (RCC) who developed recall pneumonitis on the first cycle of systemic sunitinib treatment is reported here. A 65-year-old man with RCC and bone metastasis underwent radiation therapy on his thoracic vertebrae (Th5-8) with a total dose of 24 Gy. Sunitinib (37.5 mg) was started 14 days after completing the radiation therapy. On the 14th day of sunitinib treatment, the patient developed progressive fever with worsening of dyspnea and general weakness. Treatment with pulse administration of prednisolone 1,000 mg for 3 days was initiated. Thereafter, the symptoms and the radiological findings regarding the interstitial filtration gradually improved over 7 days.

**Conclusion:**

To our knowledge, this is the first report of early onset recall pneumonitis during sunitinib therapy. At present, how sunitinib interacts with radiation therapy remains unclear. The possibility that tyrosine kinase inhibitor therapy, including with sunitinib, after radiation therapy may lead to adverse effects should be kept in mind.

## Background

Renal cell cancer (RCC) is the most lethal of the urological malignancies, and its incidence is currently increasing [1]. Recently, several new targeted agents were introduced in the clinical treatment of metastatic RCC. One of these agents, sunitinib, is an orally administered tyrosine kinase inhibitor with multiple targets, including vascular endothelial growth factor receptor (VEGFR)-1, VEGFR-2, VEGFR-3, platelet-derived growth factor receptor (PDGFR)-α, and PDGFR-β. Sunitinib is one of the most widely prescribed drugs for the treatment of metastatic RCC [2]. The adverse effects of sunitinib include fatigue, bone marrow suppression, hand-foot syndrome, stomatitis, hypertension, and hypothyroidism [3]. Interstitial lung disease is a rare but serious complication of targeted therapy with tyrosine kinase inhibitors, including sunitinib. The case of a patient with metastatic RCC who developed recall pneumonitis on the 14th day of systemic sunitinib treatment is described here. There is currently only one report of recall pneumonitis during sunitinib therapy in the literature [4]. The time of onset in that case was quite different from that in the present case.

## Case presentation

**Figure 1:**
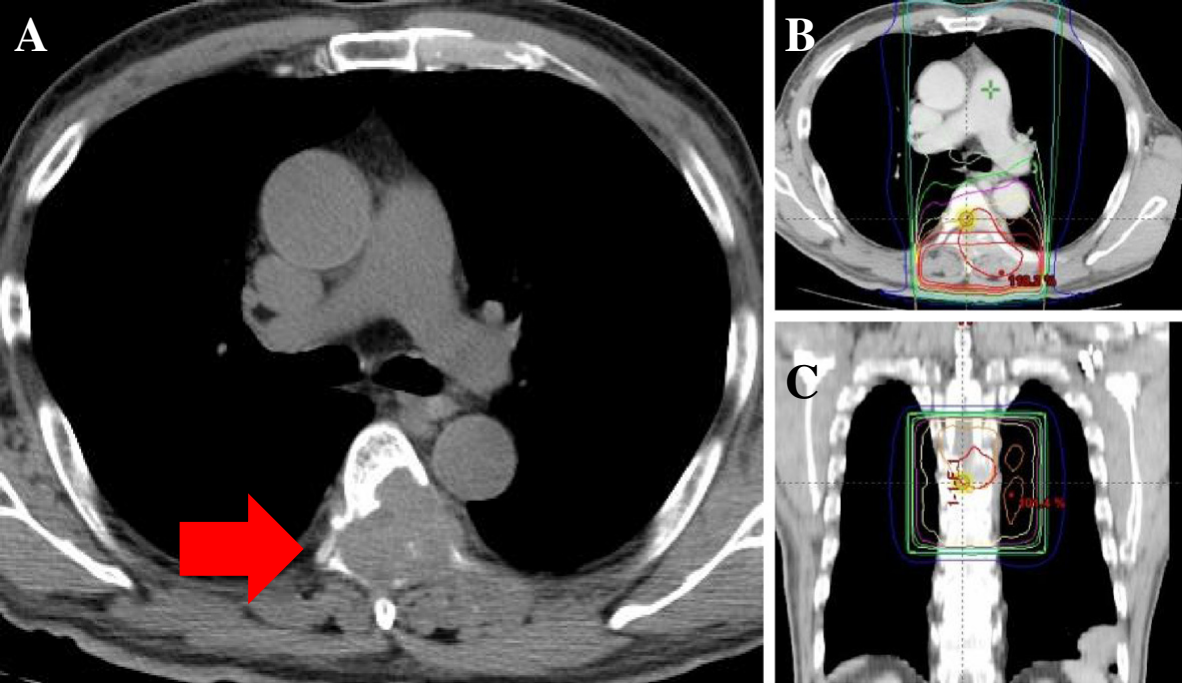
**The patient underwent radiation therapy for spinal bone metastasis. **The spinal compression from the RCC bone metastasis (**A**). The irradiated region is shown in the horizontal section (**B**) and the coronal section (**C**).

**Figure 2:**
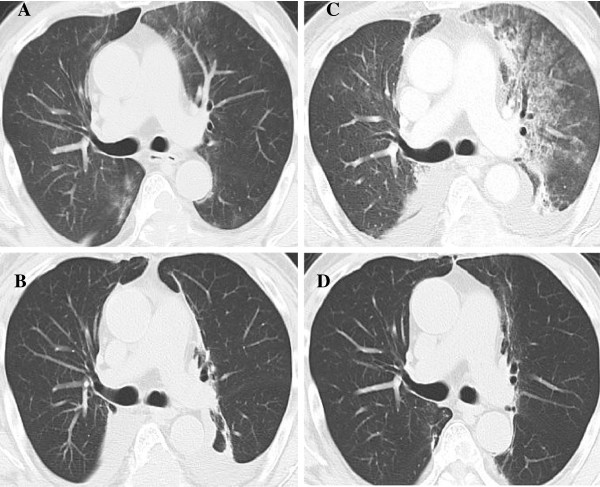
**Radiation recall pneumonitis induced by sunitinib. **On the 14th day of sunitinib induction, bilateral lung consolidation appeared (**A**). During follow-up without sunitinib, consolidation developed along with progressive fever (**B**). After steroid pulse therapy, the CT findings revealed that the interstitial filtration was localized in the previously irradiated area (**C**). No evidence of relapse of recall pneumonitis during sorafenib treatment was observed (**D**).

A 65-year-old man with clear cell RCC and lung metastasis was initially treated by right nephrectomy on November, 2011 (pT2b, N0, M1). On January, 2012, during the follow-up period, bilateral lower limb paralysis occurred due to spinal compression from RCC bone metastasis (Figure 1A). He underwent radiation therapy on his thoracic vertebrae (Th5-8) with a total dose of 24 Gy that was divided into six fractions (Figure 1B and C). Sunitinib (37.5 mg) was started 14 days after completing the radiation therapy. On the 14th day of sunitinib treatment, the patient complained of high fever and the chest CT disclosed bilateral lung consolidation (Figure 2A). At this time, blood culture examinations did not reveal any significant bacteria or fungi, and serum levels of anti-cytomegalovirus antibody, pulmonary surfactant protein-D, and sialylated carbohydrate antigen KL-6 were within the normal range. Although he was observed without sunitinib administration, the patient developed progressive fever with worsening of dyspnea and general weakness (Figure 2B). Treatment with pulse administration of prednisolone 1,000 mg for 3 days was initiated along with antifungal agent (voriconazole) and antibiotic (meropenem hydrate) treatment. After the steroid pulse therapy, the symptoms gradually improved over 7 days. The radiological findings revealed that the interstitial filtration was localized within the previously irradiated area (Figure 2C). Therefore, the patient was diagnosed with radiation recall pneumonitis that was induced by sunitinib. During the treatment, serial sputum specimen and blood culture examinations did not reveal any significant bacteria or fungus. The patient recovered from the interstitial lung disease completely during the 2 months of follow-up (Figure 2D). Currently, he is undergoing targeted therapy with another tyrosine kinase inhibitor, sorafenib, with no evidence of relapse of the tyrosine kinase inhibitor-associated recall pneumonitis.

## Discussion

The radiation recall phenomenon has been reported in the skin, lung, and mucosa [5-7]. Causal agents have been reported to be cytotoxic agents, including actinomycin D, gemcitabine, taxanes, and anthracyclines, and the estrogen receptor antagonist tamoxifen [5-7]. In most cases, dermatitis is reported. Radiation recall pneumonitis appears to be relatively rare. The mechanism of the recall response remains to be clarified. It has been proposed that it is the result of the inhibition of cellular recovery by cytotoxic agents after damage caused by radiation [6-8]. It is possible that prevention of angiogenesis by sunitinib reduces the recovery from the radiation-induced cell damage. Alternatively, radiation injury may induce hypersensitivity to sunitinib [6-8]. Alternatively, radiation injury may induce hypersensitivity to sunitinib [6-8].

A case of interstitial pneumonitis during targeted therapy with sunitinib is reported here. Pulmonary damage commonly occurs with doses more than 30 Gy and usually develops 1–6 months after cessation of radiation therapy to the lung. In the present case, the total dose of the radiation that was administered to the patient was only 24 Gy. Since the volume of lung that received more than 20 Gy (V20) was only 2% (Figure 1C) and the patient had received radiation therapy within one month before the onset of the pneumonitis, it was considered to be unlikely to be radiation-induced pneumonitis. Therefore, it was concluded that the pneumonitis was recall pneumonitis induced by sunitinib. There is currently only one report of recall pneumonitis during sunitinib therapy [4]. In that report, a female patient with RCC developed pneumonitis more than 6 months after sunitinib induction and the completion of radiation therapy (30 Gy: 5 fraction) [4]. Her pneumonitis resolved completely when the dose of sunitinib was reduced (from 50 mg/day to 37.5 mg/day); steroids were not administered [4]. Thus, this case differs from the present case in terms of the time of onset, the radiation dose that was administered, and the severity of the pneumonitis.

Although how sunitinib interacts with radiation therapy remains unclear, this interaction may lead to synergistic toxicities. There has been a report of lethal small bowel perforation after radiation for lumbar metastasis during sorafenib administration; moreover, the case of a lethal bronchial fistula that occurred after radiation for mediastinum lymph node metastasis during sunitinib therapy was published [9,10]. The current knowledge about the combination of tyrosine kinase inhibitors with radiation therapy has been reviewed by Niyazi *et al*. [7]. At present, it seems that none of the tyrosine kinase inhibitors have been approved for simultaneous use with radiation therapy.

## Conclusions

In conclusion, a case of early onset recall pneumonitis during targeted therapy with sunitinib was reported here. At present, how sunitinib interacts with radiation therapy remains unclear. The possibility that tyrosine kinase inhibitor therapy, including with sunitinib, after radiation therapy may lead to adverse effects should be kept in mind.

## Consent

Written informed consent was obtained from the patient for publication of this Case report and any accompanying images. A copy of the written consent is available for review by the Series Editor of this journal.

## Abbreviations

RCC: Renal cell cancer; VEGFR: Vascular endothelial growth factor receptor; PDGFR: Platelet derived growth factor receptor.

## Competing interests

The authors declare that they have no competing interests.

## Authors’ contributions

Conception and design: TY; Manuscript writing: TY; Final approval: SK, GS, SY, KK, KM, TK, SH, and JY; Pathological explorations: NI; Patient’s management: TY, SK, GS, SY, JY; All authors read and approved the final manuscript.

## Pre-publication history

The pre-publication history for this paper can be accessed here:

http://www.biomedcentral.com/1471-2407/13/3/prepub
